# Sneaking into Dentistry

**Published:** 1885-03

**Authors:** 


### SNEAKING INTO DENTISTRY.

Eternal vigilance seems to be the price that the dentists of the State of New York must pay for their law. Last year a bill was introduced in the interest of some dentist who had more political influence than professional knowledge, and who thought it would be easier to lobby his way into the profession than to study for it. Hence, instead of coming by the way of a reputable college, he endeavored to sneak in through the back door of a complaisant legislature. It was by the merest accident it was discovered that a bill for the reopening of the registry was actually upon its passage, and in another hour would have become law. This year, the same trick of endeavoring to make a dentist by legislation instead of by education was tried again. But Dr. C. F. Wheeler, of Albany, warned by the narrow escape of last year, was on the watch. So carefully was the matter conducted that he did not discover the bill until the very last moment. Aided by others, he went before the committee to whom the bill was referred, and secured a postponement of action. The law committee of the State Society was informed by telegraph, and Dr. William Carr, its chairman, hurried to Albany. A telegram from him, just as this form goes to press, conveys the gratifying information that the bill has been strangled in committee.

